# Acute Myocardial Infarction in a Patient With Focal Segmental Glomerulosclerosis Tip Lesion: Hit With a Double Whammy

**DOI:** 10.7759/cureus.48860

**Published:** 2023-11-15

**Authors:** Elenjickal Nikhil John, Anand Chellappan, Gunjan Ghodeshwar, Alok Sharma, Sachin R Chaudhari

**Affiliations:** 1 Department of Nephrology, All India Institute of Medical Sciences, Nagpur, Nagpur, IND; 2 Department of Cardiology, All India Institute of Medical Sciences, Nagpur, Nagpur, IND; 3 Department of Renal Pathology, Dr. Lal PathLabs/National Reference Lab, New Delhi, IND; 4 Department of Pathology, All India Institute of Medical Sciences, Nagpur, Nagpur, IND

**Keywords:** oral glucocorticoids, hypercoagulability, nephrotic syndrome, myocardial infarction, focal segmental glomerulosclerosis

## Abstract

Nephrotic syndrome is associated with venous and arterial thrombotic complications and is related to the imbalance between pro-thrombotic and anti-thrombotic factors. With an underlying nephrotic syndrome, arterial thromboses are infrequent, and coronary artery thromboses are much rarer. We present the case of a young male, with nephrotic syndrome, who suffered an acute anterior wall ST-segment elevation myocardial infarction. He was subsequently diagnosed to have focal segmental glomerulosclerosis (FSGS)-tip lesion. The patient was successfully managed with thrombolysis, steroids, anticoagulation, antiplatelets, and statins.

## Introduction

Nephrotic syndrome is associated with a hypercoagulable state. In addition to venous thromboembolic events, nephrotic syndrome is associated with an increased risk of arterial thrombotic events. While venous thrombosis is common in adults, arterial thrombosis is seen mainly in children [1]. Acute coronary syndrome is an exceedingly rare, yet life-threatening complication of adult-onset nephrotic syndrome. The possible underlying mechanisms include hypercoagulability and/or platelet hyperactivity, atherosclerosis, and drug treatment [2]. Herein, we present the case of a young man with nephrotic syndrome who developed acute anterior wall ST-segment elevation myocardial infarction. He was thrombolysed and started on anticoagulation, antiplatelets, and statins. He improved well and attained remission with glucocorticoids. A kidney biopsy done later revealed a focal segmental glomerulosclerosis tip variant.

## Case presentation

A 29-year-old man presented with complaints of swelling of lower limbs and facial puffiness for the last three months. He denied any symptoms of dysuria, passage of cola-colored urine, joint pains, oral ulcers, or rashes. There was no fever, skin infection, or sore throat preceding the complaints. Family history was negative for any renal or cardiovascular disease. He did not have any other comorbidities. He did not have any addictions. There was no history of taking non-steroidal anti-inflammatory drugs, diuretics, or anabolic steroids. He admitted taking native indigenous medicines for few days after the onset of swelling. The nature of the medications is not known. He also complained of shortness of breath on exertion. The patient was admitted with the above-mentioned complaints and was found to have significant pedal edema and reduced air entry on both sides on chest auscultation. In view of volume overload, he was started on intravenous diuretics. A chest x-ray was done which revealed moderate pleural effusion (Right>Left). Laboratory investigations (Table 1) revealed a low serum albumin- 1.15 g/dL and his urine routine microscopy showed the presence of 3+protein, 3-4 RBCs, and 10-12 WBCs/hpf with occasional cellular and granular casts. He had acute kidney injury with oliguria at presentation with the peak serum creatinine being 2.98 mg/dL that subsequently improved to 1.1 mg/dL at discharge. A urine spot protein creatinine ratio was 680mg/g. Twenty-four-hour urine protein estimation was not done. He also had dyslipidemia. He was diagnosed as a case of nephrotic syndrome and was planned for a kidney biopsy after stabilization.

**Table 1:** Laboratory investigations at presentation SGOT: Serum glutamic oxaloacetic transaminase; SGPT: serum glutamic pyruvic transaminase; HDL: high-density lipoprotein; LDL: low-density lipoprotein; ANA: antinuclear antibody

Sr. No.	Investigation	Value	Reference Range
1	Hemoglobin	11.1g/dL	13-17g/dL
2	Total leucocyte count	8.20x10^3^/µL	4-10 x10^3^/µL
3	Platelet count	281 x10^3^/µL	150-450 x10^3^/µL
4	Blood urea	88.1 mg/dL	15-39mg/dL
5	Serum creatinine	2.98 mg/dL	0.6-1.3mg/dL
6	Serum albumin	1.15 g/dL	3.2-4.5g/dL
7	Serum SGOT/SGPT	38/20 U/L	0-45 U/L
8	Urine routine microscopy	Protein 3+	Nil
		3-4 RBCs/hpf	Nil
		10-12 WBCs/hpf	Nil
		Occasional cellular and granular casts seen	Nil
9	Urine spot protein- creatinine ratio	680 mg/g	<200mg/g
10	Serum total cholesterol	650 mg/dL	<200mg/dL
11	Serum HDL cholesterol	33.3 mg/dL	>55mg/dL
12	Serum LDL cholesterol	555 mg/dL	<100mg/dL
13	Serum triglycerides	306 mg/dL	<150mg/dL
14	Viral Markers (HIV, HbsAg, HCV) (by Immunochromatography )	Negative	Negative
15	Anti PLA2R	<2 RU/ml	<14RU/mL
16	ANA	Negative	Negative
17	C3	90.6mg/dL	90-180mg/dL
18	C4	31.1mg/dL	10-40mg/dL
19	Urine culture	No growth	No growth
20	Serum procalcitonin	< 0.05 ng/mL	<0.5 ng/mL

**Figure 1:**
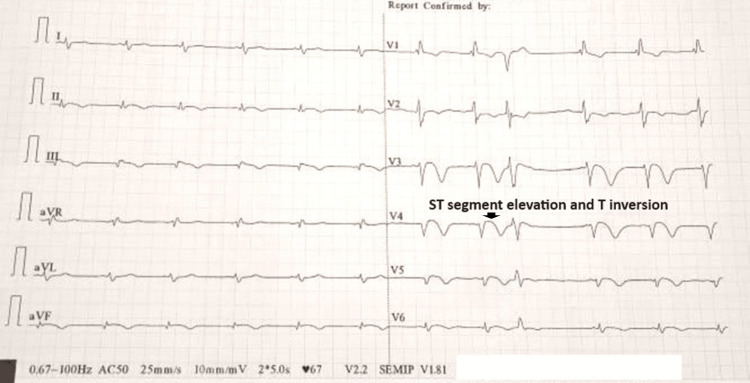
Electrocardiogram at the time of chest pain

During hospitalization, he complained of retrosternal chest pain, and an electrocardiogram (ECG) was done, which showed ST-T wave changes along anterior chest leads (Figure 1). Troponin I was found to be elevated (>0.04ng/mL). There was no hemodynamic compromise. A cardiologist consultation was sought for, and a subsequent 2D transthoracic echocardiogram showed regional wall motion abnormality along the left anterior descending (LAD) artery territory. With an ongoing acute coronary event, the patient was thrombolysed with streptokinase. Chest pain settled after thrombolysis. He was empirically started on steroids in view of nephrotic syndrome with suspected thrombotic complications. Anti PLA2R antibody and ANA were sent, both of which came out to be negative, and serum complement levels were normal.

**Figure 2:**
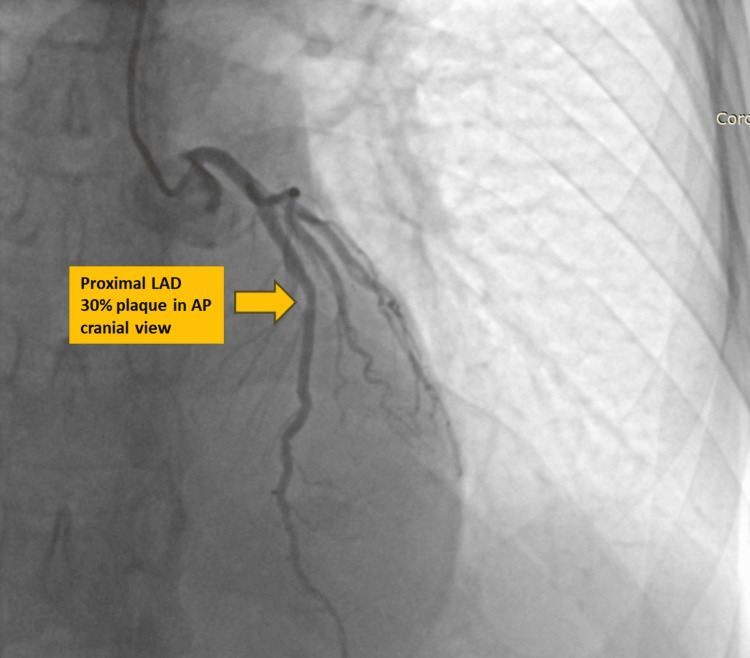
Coronary angiogram showing 30% plaque in the proximal left anterior descending (LAD) artery

After his renal functions stabilized, he underwent a coronary angiogram, which revealed the presence of a recanalized Left anterior descending artery, with the proximal mid-junction region showing 30% plaque, and the diagonal branch showing 90% stenosis (Video 1 and Figure 2). The patient was treated with unfractionated heparin, dual antiplatelets, and statins and observed. Kidney biopsy was deferred in view of the need for starting antiplatelets and anticoagulants. The patient improved with the aforementioned line of treatment, and edema subsided. He was discharged and was followed up in the outpatient clinic thereafter.

**Video 1:** Coronary angiogram

**Figure 3:**
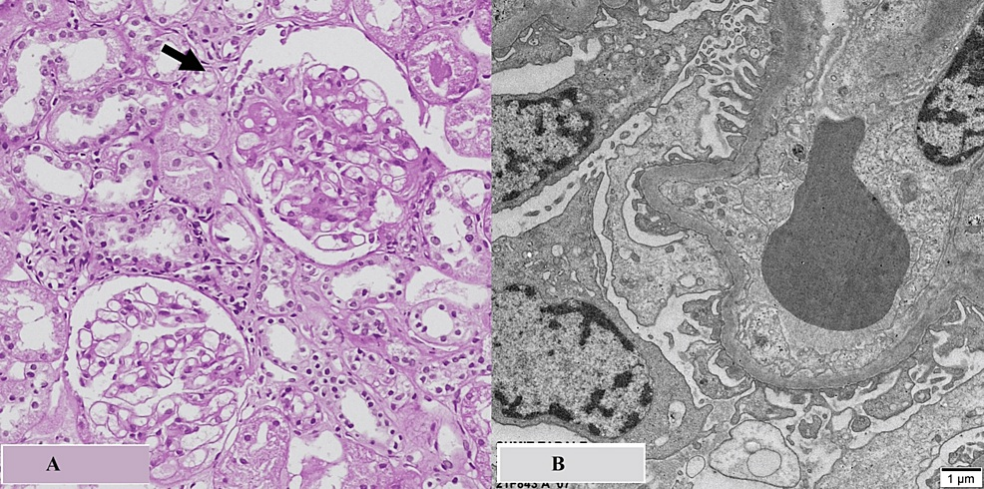
Kidney biopsy (A) H&E section from kidney biopsy at 200x magnification shows two glomeruli. The upper glomerulus near arrow shows tip lesion with sclerosis and adhesion of capillary loop to capsule near origin of proximal convoluted tubule and presence of intracapillary foam cells in lumen. (B) Transmission electron microscopy section stained with Lead citrate and uranyl acetate shows diffuse effacement of foot processes of podocytes in non-sclerotic capillary loops. Microvillous transformation is visible.

Four months later, the patient was readmitted for kidney biopsy. After taking an opinion from the cardiologist, the antiplatelets were kept on hold temporarily to avoid bleeding. He underwent an ultrasound-guided percutaneous kidney biopsy, which was uneventful. The kidney biopsy revealed the presence of segmental tuft sclerosis, with foamy cellular changes along the tip region of the capillary tuft. There was no evidence of any tuft necrosis or crescents (Figure 3). The immunofluorescence study revealed segmental trapping of C3, and the rest of the immunological markers (IgA, IgG, IgM, C1Q, Kappa, and Lambda) were negative. Electron microscopy revealed diffuse effacement of podocyte foot processes.

He was diagnosed with a case of primary focal segmental glomerulosclerosis (FSGS)-tip lesion, as per the histomorphologic classification. Steroids were optimized as per the KDIGO 2021 guidelines, and subsequently, antiplatelets were re-initiated. His diet was modified for a salt-restricted heart-healthy diet. Currently, the patient is doing well on follow-up and is in clinical remission, with reduction in his proteinuria, improvement in serum albumin, and no edema. Table 2 shows the trends of important laboratory parameters. 

**Table 2:** Trends of important laboratory parameters

Sr. No.	Investigation	At Presentation	Day 8	Day 21	Follow up: 3 months	Follow up: 10 months	Last follow up: 27 months	Reference Range
1	Blood Urea (mg/dL)	88.1	115.3	34.4	30.4	24.9	Not done	15-39
2	Serum Creatinine (mg/dL)	2.98	1.71	0.64	0.74	0.98	Not done	0.6-1.3
3	Serum Albumin (g/dL)	1.15	1.33	1.4	3.49	4.68	4.86	3.2-4.5
4	Urine Spot Protein- Creatinine Ratio (mg/g)	680	Not done	Not done	Not done	323	109	<200
5	Serum Calcium (mg/dL)	Not done	6.8 (Day 12)	6.0	Not done	9.5	Not done	8.4-10.2

## Discussion

**Figure 4:**
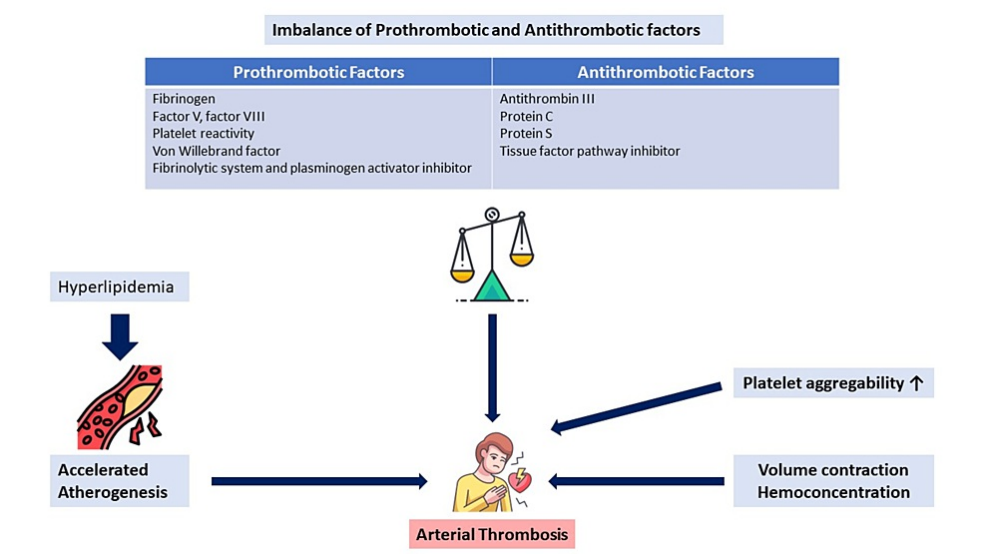
Mechanisms underlying arterial thrombosis in nephrotic syndrome

Nephrotic syndrome is known to be associated with thrombotic complications. The relative risk for arterial and venous thrombotic events is higher than that seen in the general population, with venous thromboembolism rates ranging from 2% in children to as high as 45% in adults and a relative risk of arterial thromboembolism ranging from 1 to 5.5 [3]. It has been found to be related to the serum albumin level and the underlying cause of nephrotic syndrome. Among all causes of nephrotic syndrome, thrombotic complications are most common in membranous nephropathy followed by FSGS [4]. The underlying mechanisms are poorly understood; however, the possible causes postulated include raised levels of fibrinogen, increased blood viscosity, hemoconcentration, thrombocytosis, platelet activation and aggregation, and reduced levels of antithrombin III and protein C (Figure 4).

FSGS is characterized by glomerular tuft sclerosis. “Focal” refers to involvement of few glomeruli, and “segmental” implies the involvement of a portion of the glomerulus. On electron microscopy, diffuse podocyte foot process flattening is often observed. Much information has been gained in the past few decades and with the understanding of the pathogenesis of the disease, FSGS is classified as primary, secondary, genetic and of undetermined significance [5]. Histologically, primary FSGS is divided into 5 subtypes by the Columbia classification- Perihilar, Collapsing, Tip, Cellular and not otherwise specified (NOS) [6]. Traditionally, the tip lesion type of FSGS has been considered to be associated with the best prognosis, with respect to response to treatment and earlier remission rates [7]. However, the lesion may be not as benign as it seems and may be associated with worsening proteinuria, deteriorating renal functions, and difficulty in achieving remission rates [8].

As described by Li et al. [9], the prevalence of venous thrombotic events in FSGS is approximately 10%. Arterial thrombotic complications are even fewer, and most are cited as case reports. Coronary thrombotic events with an underlying nephrotic syndrome are much more rarely seen and reported [10,11]. The increased risk of arterial thrombotic events has been attributed to increased platelet activation and aggregation which happens in nephrotic syndrome. Lee et al. did a retrospective study to evaluate the risk of cardiovascular events relative to that of end-stage renal disease in patients with primary membranous nephropathy [12]. Their data revealed two patterns of arterial thrombotic events associated with nephrotic syndrome. The ‘early events’ typically reported less than two years after diagnosis were associated with severe proteinuria, hypoalbuminemia, or both. The ‘late events’ (beyond two years after diagnosis) may be attributable to classic pathogenetic mechanisms and risk factors (age, diabetes, and previous history of arterial thrombotic events) of atherosclerosis. Hofstra et al. suggest an algorithm for the use of antiplatelets in patients with nephrotic syndrome and increased arterial thrombotic risk [13]. Our patient was a young adult male who developed an early arterial thrombotic event within three months of diagnosis and it was accompanied by severe hypoalbuminemia and proteinuria. The sub-nephrotic range proteinuria as estimated by the Urine Spot protein creatinine ratio could possibly be explained by the acute kidney injury at the time of presentation. A 24-hour urine protein estimation was not done. A close differential to acute myocardial infarction is Stress cardiomyopathy. The clinical presentation of stress cardiomyopathy may be indistinguishable from that of an acute myocardial infarction. The absence of an emotional stressor, presence of an elevated serum Troponin I level, occurrence of regional wall motion abnormality confined to LAD territory on echocardiography, and the improvement in chest pain after thrombolysis favor the diagnosis of an acute myocardial infarction in our case. A successful and timely thrombolysis may potentially account for the absence of a clearly evident thrombus in the coronary angiogram. An exhaustive literature search showed few case reports of myocardial infarction in the presence of an underlying nephrotic syndrome. Hopp et al. reported a case of myocardial infarction (MI) in a seven-year-old child with steroid-resistant nephrotic syndrome and hyperlipidemia, diagnosed later with minimal change disease [14]. Similarly, Meyer and colleagues [15] reported a case of anterior wall MI in a 37-year-old female with nephrotic syndrome, with underlying minimal change disease. In a retrospective case series (2008-2016) by Xie and colleagues [16], eight patients with nephrotic syndrome, suffered an acute coronary event, requiring either percutaneous coronary intervention or thrombus aspiration. Membranous nephropathy was the most common cause of nephrotic syndrome in their series. Recently, Sjuls et al. [17] reported a 26-year-old male patient, a diagnosed case of FSGS, presenting with anterior wall STEMI (ST-segment elevation myocardial infarction). A subsequent coronary angiogram revealed the presence of a LAD thrombus, which was managed with thrombectomy and local intracoronary thrombolysis. He was treated with antiplatelets, high-intensity statins, and warfarin anticoagulation. The authors used a novel treatment for hyperlipidemia by employing PCSK 9 inhibitors (evolocumab) and SGLT-2 inhibitor (empagliflozin) and noted a fair improvement in the serum albumin level and decrease in the proteinuria (from 5.1 g/day to 2g/day).

## Conclusions

Acute coronary syndrome is a rare complication of adult-onset nephrotic syndrome. This case highlights that nephrotic syndrome is associated with increased risk for serious arterial thrombotic events including acute coronary syndrome. FSGS tip lesion though considered to have the best prognosis may be associated with increased thrombotic risk depending upon the associated risk factors and presentation. Arterial thrombotic events may present early or late after diagnosis and the risk factors may differ between the two presentations. Hence, a thorough evaluation, early recognition of risk factors, and prompt treatment can help prevent catastrophic thrombotic events.
